# Breaking Genetic Barriers: Understanding the Limits of Horizontal Gene Transfer

**DOI:** 10.1093/gbe/evad102

**Published:** 2023-06-15

**Authors:** Casey McGrath

The recognition of the phenomenon known as horizontal (or lateral) gene transfer (HGT/LGT) revolutionized our understanding of evolutionary mechanisms. Unlike the conventional vertical transmission of genes from parent to offspring, HGT involves the exchange of genetic material laterally, across species boundaries. This process is a major contributor to microbial evolution, accounting for 10–20% of the protein-coding genes in most bacterial genomes, while HGT is less prevalent among eukaryotes. Through HGT, bacteria and archaea can acquire new traits, ranging from antibiotic resistance to metabolic capabilities, which enhances their ability to adapt to changing environments. In a study titled “Empirical evidence that complexity limits horizontal gene transfer”, researchers from the University of North Carolina, led by Christina Burch and Corbin Jones, investigated the factors that influence the ability of individual genes to be transferred into a new recipient bacterial strain via HGT (Burch et al. 2023). Their study reveals that a gene's transferability is affected by several factors, including its sequence divergence from the recipient and how many interactions partners the resulting protein has (i.e., its connectivity). Moreover, a gene's divergence and connectivity interact to further influence its transferability.

While previous studies have observed a relationship between gene transferability and protein connectivity, scientists have puzzled over the mechanism underlying this link. Two potential hypotheses have been suggested: the Balance Hypothesis and the Complexity Hypothesis. The Balance Hypothesis suggests that newly transferred genes may result in gene dysregulation by upsetting the balance between expressed proteins, while the Complexity Hypothesis proposes that newly transferred genes may fail to engage in normal protein-protein interactions. Importantly, while the divergence between the donor and recipient strains should not affect the former process, it is expected to impact the latter, as more divergent proteins are more likely to experience protein–protein interaction failure. Thus, the tendency of divergence to magnify the effect of connectivity on transferability can be used to distinguish between these two hypotheses.

To untangle the impact of these mechanisms on HGT, Burch et al. (2023) reanalyzed old data in a new way. Some of the earliest bacterial and archaeal genomes were sequenced using a method in which the genome of interest was fragmented and each fragment was incorporated onto a bacterial plasmid (a small piece of circular DNA). The plasmids were then cloned into *Escherichia coli*, which would reproduce and generate more copies of the plasmids for subsequent shotgun sequencing and assembly. Over fifteen years ago, Sorek et al. (2007) recognized that these libraries could be used to assess the transferability of genes via HGT; they found that certain genes were “unclonable”, meaning that they could not be transferred on a plasmid from the host-microbe to *E. coli*. Burch et al. (2023) used a similar data set that included 70 bacteria and 4 archaea but performed a quantitative analysis of transferability, using sequencing coverage as a proxy to indicate how easily each gene was transferred into *E. coli*.

Initially, Burch and her collaborators noticed significant biases in the data, with both the length and position of a gene affecting its coverage. According to Burch, “The very first thing we noticed was that the read coverage was dramatically higher at the origin of genome replication than at the terminus in some of the shotgun libraries. Of course, that makes sense if you know that actively growing bacteria initiate lots of replication forks at the origin. With this knowledge of bacterial physiology in hand, we could infer that some of the shotgun libraries were made using genomes isolated from actively growing cells, whereas others were made using genomes isolated from cells that were not actively growing. Although this particular aspect of bacterial physiology was not relevant to the question we set out to answer, our ability to see its effect on the shotgun library data reassured me at an early stage that the biological signals we were interested in studying might also be detectable.” In other words, these data suggested that the researchers would have the power to detect patterns among the variables that influence HGT.

After correcting for biases related to bacterial physiology (i.e., frequent initiation of replication in actively growing cells), the authors investigated the relationship between gene transferability (as estimated by sequencing coverage) and several factors that may affect HGT, including gene function, protein connectivity, the divergence between the donor species and *E. coli*, and the expression level of the native gene in *E. coli*. Importantly, they found a significant interaction between divergence and connectivity, supporting the Complexity Hypothesis and suggesting that the ability of a transferred gene to engage in normal protein-protein interactions plays a key role in the success or failure of HGT.

In addition to these findings, an important contribution of this study was the development of a statistical test capable of evaluating the Complexity Hypothesis. Burch notes, “Prior to this work, the Complexity Hypothesis had been described only using verbal arguments. I think it was an important step forward to translate the hypothesis into a specific statistical test. The fact that we could then conduct the statistical test on existing genomic data was icing on the cake. We are grateful to the Sorek et al. (2007) team for leading the way.”

One caveat of this analysis is that all the genes studied were on the plasmids (i.e., extrachromosomal DNA) used to transfer them into the recipient cell. Different dynamics may be observed when genes are transferred directly onto bacterial or archaeal chromosomes. “Ultimately, we would like to understand better the consequences of incorporating transferred genes into recipient genomes,” says Burch. “Modern genome sequencing technology makes it possible to investigate that question using microbial evolution experiments, and a few have been done, but a lot more data are needed.” Moreover, the current analysis was necessarily limited to genes that were already present in the *E. coli* genome. “We would also like to understand better the horizontal transfer of new or accessory genes that are not already present in recipient cells,” continues Burch. “Those genes are not relevant to the Complexity Hypothesis, so that investigation remains for future work.”
